# Numerical Built-In Method for the Nonlinear JRC/JCS Model in Rock Joint

**DOI:** 10.1155/2014/735497

**Published:** 2014-02-11

**Authors:** Qunyi Liu, Wanli Xing, Ying Li

**Affiliations:** Institute of Mineral Resources, Chinese Academy of Geological Sciences, Beijing 100037, China

## Abstract

The joint surface is widely distributed in the rock, thus leading to the nonlinear characteristics of rock mass strength and limiting the effectiveness of the linear model in reflecting characteristics. The JRC/JCS model is the nonlinear failure criterion and generally believed to describe the characteristics of joints better than other models. In order to develop the numerical program for JRC/JCS model, this paper established the relationship between the parameters of the JRC/JCS and Mohr-Coulomb models. Thereafter, the numerical implement method and implementation process of the JRC/JCS model were discussed and the reliability of the numerical method was verified by the shear tests of jointed rock mass. Finally, the effect of the JRC/JCS model parameters on the shear strength of the joint was analyzed.

## 1. Introduction

The Mohr-Coulomb linear model is generally used to study the characteristics of rock mass strength [1–3]. Lin et al. [1] used numerical algorithm to simulate the mechanical behavior of a layered rock mass under true triaxial compression by Mohr-Coulomb linear model. Most calculation software are based on the Mohr-Coulomb model, which represents rock strength with cohesion *c* and internal friction angle *ϕ* [4–7]. However, the joint surface is widely distributed in the rock, thus leading to the nonlinear characteristics of rock mass strength and limiting the effectiveness of the linear model in reflecting characteristics [8]. Therefore, scholars introduced nonlinear models, such as the Hoek-Brown model [9, 10] and JRC/JCS model [11–13], which can describe rock mass strength. Halakatevakis and Sofianos [14] investigated the Hoek-Brown criterion analytically through an extended plane of weakness theory. Babanouri et al. [13] developed extended Barton's shear failure criterion for rock joints to consider the effect of various paths of normal loading/unloading before shearing and overconsolidation ratio in a fracture. Park and Song [15] produced a rough joint with a joint roughness coefficient (JRC) value ranging from 10 to 20 in an intact sample by defining the jointcontacts along a predefined joint surface. Studies have contributed significantly to the nonlinear description of rock mass strength. However, the JRC/JCS model, which was proposed by Barton et al. on the basis of a large number of shear tests on jointed rock mass, is generally believed to describe the characteristics of joints better than other models [11, 16]. The JRC/JCS model is mainly studied by theoretical and experimental approaches and not by numerical approaches. On the basis of the above considerations, this paper established the relationship between the parameters of the JRC/JCS and Mohr-Coulomb models. Thereafter, the numerical implement method and implementation process of the JRC/JCS model were discussed and the reliability of the numerical method was verified by the shear tests of jointed rock mass. Finally, the effect of the JRC/JCS model parameters on the shear strength of the joint was analyzed.

## 2. Theoretical Derivation

### 2.1. Relationship between the Parameters of the JRC/JCS Model and Mohr-Coulomb Model

The JRC/JCS model was formulated as follows [11]:

(1) $$\begin{array}{c} \tau =\sigma_{n}\tan\left[\phi_{b}+\text{JRC}\;\text{lo}{\text{g}}_{10}\left(\frac{\text{JCS}}{\sigma_{n}}\right)\right], \end{array}$$

where *τ* is the rock shear strength; *σ*_*n*_ is the joint normal stress; *ϕ*_*b*_ is the basic friction angle of the rock, which can be fixed at 30°; JRC is the joint roughness coefficient, which has a value that is related to the joint rough shape; JCS is the rock compressive strength and has a slight effect on shear strength under low stress conditions. The influence of JCS increases with increasing normal stress.

The Mohr-Coulomb model was formulated as follows:

(2) $$\begin{array}{c} \tau_{c}=\sigma_{n}\tan\phi +c. \end{array}$$

Equation (2) shows that the strength of the joint surface depends on the following strength parameters: cohesion *c* and internal friction angle *ϕ* in the Mohr-Coulomb model [17]. The derivation of (2) is as follows:

(3) $$\begin{array}{c} \tan\phi =\frac{\partial \tau }{\partial \sigma_{n}}. \end{array}$$

By integrating (1) and (3), we obtained the following:

(4) $$\begin{array}{c} \tan\phi =\frac{\partial \tau }{\partial \sigma_{n}}=f_{a}-f_{b}, \end{array}$$

where *f*_*a*_ = tan(*ϕ*_*b*_ + JRC · log (JCS/*σ*_*n*_)), *f*_*b*_ = (*π*JRC/180ln⁡10)(*f*_*a*_^2^ + 1).

The triangle transformation of (4) is as follows:

(5) $$\begin{array}{c} \phi =\arctan\left(f_{a}-f_{b}\right),\\ \sin\phi =\frac{f_{a}-f_{b}}{f_{a}^{2}+f_{b}^{2}-2f_{a}f_{b}},\\ \cos\phi =\frac{1}{f_{a}^{2}+f_{b}^{2}-2f_{a}f_{b}},\\ c=\sigma_{n}f_{b}. \end{array}$$

The formula of *f*_*a*_ became unacceptable when *σ*_*n*_ → 0, *ϕ*_*b*_ + JRC · log_10_(JCS/*σ*_*n*_) → *∞*. Therefore, Barton suggested that *ϕ*_*b*_ + JRC · log_10_JCS/*σ*_*n*_ should be smaller than 70° in the actual project. The minimum of the normal stress could be obtained by *ϕ*_*b*_ + JRC · log (JCS/*σ*_*n*_) = 70°:

(6) $$\begin{array}{c} \sigma_{\min}=10^{\left[\text{ log }(\text{JCS})(\left(70-\phi_{b}\right)/\text{JRC})\right]}. \end{array}$$

### 2.2. Relationship Analysis of the Model Parameters

**Figure 1:**
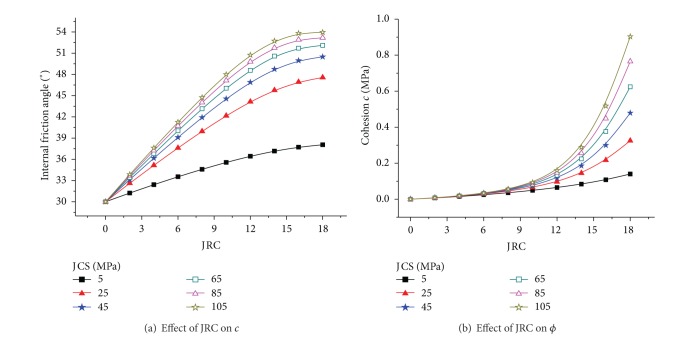
Effect of JRC.

**Figure 2:**
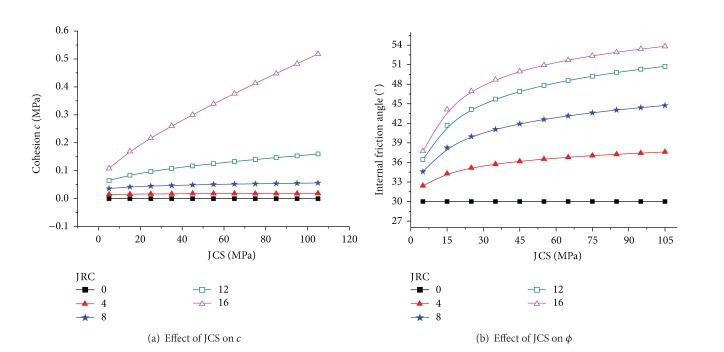
Effect of JCS.

Assuming that the rock basic friction angle was 30° and that JCS = 5 MPa to 105 MPa and JRC = 0 to 18 were changed, Figures 1 and 2 could be determined. The cohesion *c* and internal friction angle *ϕ* increase gradually with increasing JRC (Figure 1), and the slope of the relation curve of *c* and JRC increases gradually with increasing JRC. By contrast, the slope of the relation curve of *ϕ* and JRC decreases gradually with increasing JRC. The curve of the effect of JRC on the internal friction angle (Figure 1(b)) demonstrates that the internal friction angle that corresponds to the same JRC increases with increasing rock compressive strength. Furthermore, for the same increment of JCS, the increment of the internal friction angle is greater with increasing JRC. However, for the same increment of JRC, the range ability of the internal friction angle decreases with increasing JCS. This observation highlights that the effect of JRC on the internal friction angle is greater than the effect of JCS. Cohesion *c* increases linearly with increasing JCS, whereas the internal friction angle *ϕ* increases nonlinearly with increasing JCS (Figure 2). The slope of the curve of JCS and cohesion increases gradually with increasing JRC; thus, a rougher joint surface corresponds to the greater influence of JCS on cohesion (Figure 2(a)). When JRC = 0, that is, the roughness of the joint surface is high, the internal friction angle of joint surface is the basic friction angle with a value of 30° (Figure 2(b)).

## 3. Numerical Implementation

### 3.1. Numerical Computation Method

To establish the numerical computation method in the JRC/JCS model, the incremental formula of stress and deformation was obtained by elastic incremental theory:

(7) Δσ1=α1Δε1e+α2(Δε2e+Δε3e),Δσ2=α1Δε2e+α2(Δε1e+Δε3e),Δσ3=α1Δε3e+α2(Δε1e+Δε2e),

where *α*_1_ = *K* + 4*G*/3, *α*_2_ = *K* − 2*G*/3, *G* is the initial stress, and *K* is the stress increment. Δ*ε*_*i*_^*e*^ is the elastic incremental strain in the *i* direction.

The stress components could be calculated by elastic theory:

(8) $$\begin{array}{c} \sigma_{i}^{I}=\sigma_{i}^{0}+\Delta \sigma_{i},\;\left(i=1,2,3\right), \end{array}$$

where *σ*_*i*_^0^ and Δ*σ*_*i*_ are the normal stress and shear strength updated by calculation, respectively.

Thereafter, new stress components *σ*_1_^*N*^, *σ*_2_^*N*^, and *σ*_3_^*N*^ could be deduced by the theory that incremental strain Δ*ε*_*i*_ is the sum of the elastic incremental strain Δ*ε*_*i*_^*e*^ and plastic incremental strain Δ*ε*_*i*_^*p*^ under the plastic state:

(9) σ1N−σ10=α1(Δε1−Δε1p)+α2(Δε2+Δε3−Δε3p),

(10) σ2N−σ20=α1Δε2+α2(Δε1−Δε1p+Δε3−Δε3p),

(11) σ3N−σ30=α1(Δε3−Δε3p)+α2(Δε1+Δε2−Δε1p).

By integrating (7) into (11), we obtained the following:

(12) σ1N=σ1I−α1Δε1p−α2Δε3p,σ2N=σ2I−α2(Δε1p+Δε3p),σ3N=σ3I−α1Δε3p−α2Δε1p

because of

(13) $$\begin{array}{c} \sigma_{n}^{N}=\frac{1}{2}\left[\sigma_{1}^{N}+\sigma_{3}^{N}+\left(\sigma_{1}^{N}-\sigma_{3}^{N}\right)\cos2\alpha \right],\\ \tau^{N}=\frac{1}{2}\left[\left(\sigma_{1}^{N}-\sigma_{3}^{N}\right)\sin2\alpha \right], \end{array}$$

where *σ*_*n*_^*N*^ and *τ* ^*N*^ are the normal stress and shear strength updated by calculation, respectively. *α* is the angle of *σ*_1_ and normal direction of the joint surface.

### 3.2. Verification of the Numerical Method

**Figure 3:**
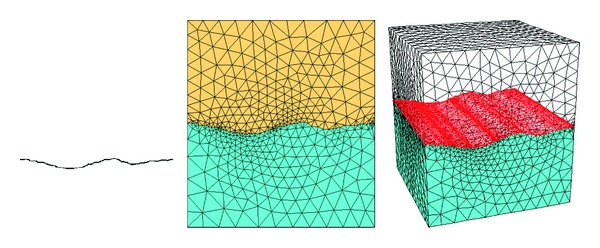
Numerical calculation model with JRC = 16.

**Figure 4:**
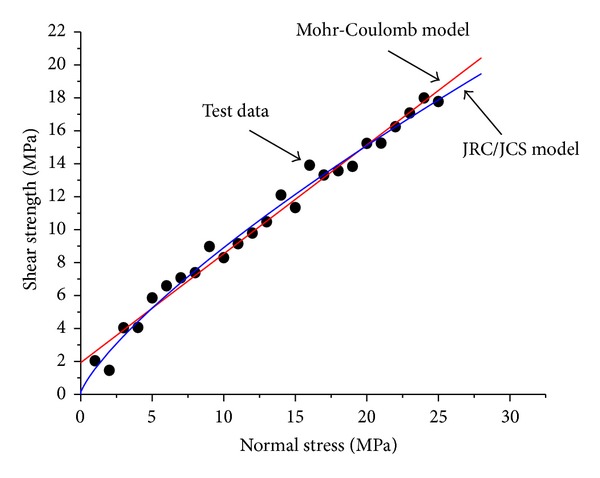
Comparison between numerical calculation and test results.

The VC++ language was used for the secondary development of the numerical calculation module of FLAC3D, and the corresponding program was developed [18–20]. During the calculation, the strain and the stress of every unit were calculated by elastic theory. Thereafter, to judge if the yield condition was achieved, the corresponding stress should be adjusted to meet the yield function by adding the JRC/JCS model. Test data that comprised the shear strength and normal stress of the joint obtained by joint shear tests in the laboratory were used to verify the correctness of the program, as shown in Table 1. The JRC = 16 is taken for calculation, and the numerical model is shown in Figure 3. As described by Lin et al. [21], FLAC3D is difficult to use for building large, complex, and three-dimensional mining models. Lin et al. [21] combined the advantages of FLAC3D in numerical calculation and those of SURPAC in three-dimensional modeling and compiled the interface program. In this paper, we used ANSYS to build the model then transformed it to FLAC3D. The comparison between the calculated and test results is shown in Table 1 and Figure 4. The joint shear strength obtained from the test increases non-linearly with increasing normal stress. Moreover, the results from the Mohr-Coulomb model and JRC/JCS model are in good agreement with the test results. However, when the normal stress of the joint surface is small, the nonlinear characteristics of the shear strength are obvious and the Mohr-Coulomb model cannot describe the nonlinear characteristics. By contrast, the JRC/JCS model can describe the characteristics well. The correlation coefficient of the results from the JRC/JCS model and test model is 0.99315, which is higher than the correlation coefficient of the results of the Mohr-Coulomb model at 0.99072. Therefore, the reliability of the numerical calculation method of the JRC/JCS model is verified. The calculations show that the parameters of the JRC/JCS model are JRC = 15.42 and JCS = 57.387 MPa, whereas the parameters of the Mohr-Coulomb model are *c* = 1.926 MPa and *ϕ* = 33.45°.

### 3.3. Analysis of Parameter Effects

**Figure 5:**
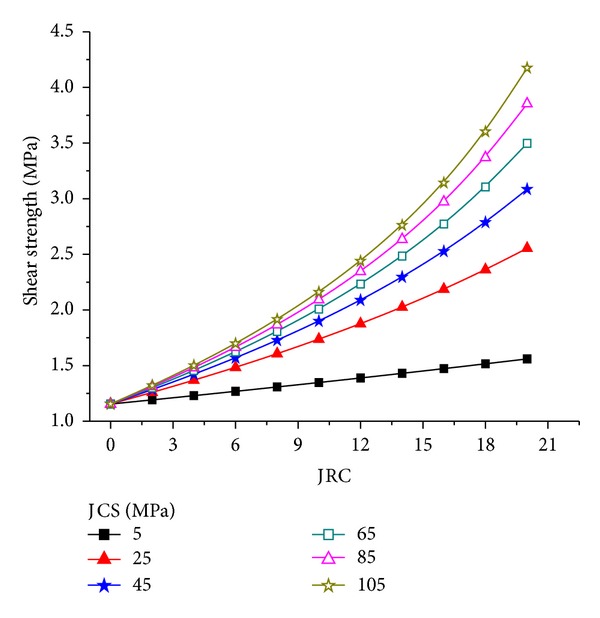
Relationship between the JRC and shear strength of the joint surface.

**Figure 6:**
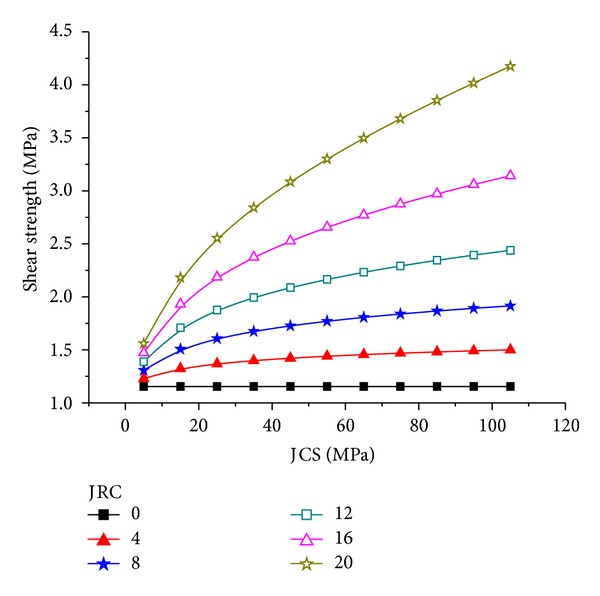
Relationship between the JCS and shear strength of the joint surface.

To study further how JRC and JCS affect the shear strength of the joint surface, *σ*_*n*_ = 2.0 MPa was fixed through calculation and the relationship of JRC and JCS with the shear strength of the joint surface was obtained (Figures 5 and 6). In Figures 5 and 6 the shear strength of the joint surface increases non-linearly with increasing JRC and JCS. The slope of the relation curve of the shear strength and JRC increases with increasing JRC, whereas the slope of the relation curve of shear strength and JCS decreases with increasing JCS. Thus, changes in JRC affect the shear strength more than changes in JCS.

## 4. Conclusions

(1) The cohesion *c* and internal friction angle *ϕ* increase gradually with increasing JRC. The internal friction angle that corresponds to the same JRC increases with increasing rock compressive strength. Furthermore, for the same increment of JCS, the increment of the internal friction angle is greater with increasing JRC. The effect of JRC on the internal friction angle is greater than the effect of JCS. Cohesion *c* increases linearly with increasing JCS, whereas the internal friction angle *ϕ* increases non-linearly with increasing JCS.

(2) The results from the Mohr-Coulomb model and JRC/JCS model are in good agreement with the test results. However, when the normal stress of the joint surface is small, the nonlinear characteristics of the shear strength are obvious and the Mohr-Coulomb model cannot describe the nonlinear characteristics. By contrast, the JRC/JCS model can describe the characteristics well.

(3) The shear strength of the joint surface increases non-linearly with increasing JRC and JCS. Changes in JRC affect the shear strength more than changes in JCS.

## Figures and Tables

**Table 1:** Validation of the numerical model.

Normal stress/MPa	Shear strength by test/MPa	Shear strength by Mohr-Coulomb model/MPa	Shear strength by JRC/JCS model/MPa
1	2.038	2.587	1.547
2	1.459	3.247	2.604
3	4.044	3.908	3.545
4	4.064	4.569	4.417
5	5.859	5.230	5.24
6	6.589	5.890	6.025
7	7.076	6.551	6.781
8	7.388	7.212	7.511
9	8.976	7.872	8.22
10	8.297	8.533	8.91
11	9.146	9.194	9.583
12	9.790	9.854	10.241
13	10.475	10.515	10.886
14	12.102	11.176	11.519
15	11.338	11.837	12.14
16	13.911	12.497	12.751
17	13.315	13.158	13.352
18	13.579	13.819	13.944
19	13.840	14.479	14.527
20	15.238	15.140	15.103
21	15.254	15.801	15.671
22	16.248	16.461	16.232
23	17.081	17.122	16.786
24	17.995	17.783	17.333
25	17.778	18.444	17.875
